# Trends in the management and coding of chronic kidney disease in Spain: cross-sectional analyses of real-life data with a 5-year interval

**DOI:** 10.1093/ckj/sfag213

**Published:** 2026-06-24

**Authors:** Rafael Santamaría, Belén Pimentel, Beatriz Palacios, Ignacio Hernández, Carlos Escobar, Manuel Gorostidi, Ana Cebrian, Roberto Alcázar

**Affiliations:** Nephrology Service, Reina Sofia University Hospital, Maimonides Institute for Research in Biomedicine of Cordoba (IMIBIC), University of Cordoba, Cordoba, Spain; BioPharmaceuticals Medical, AstraZeneca, Madrid, Spain; BioPharmaceuticals Medical, AstraZeneca, Madrid, Spain; Atrys Health, Madrid, Spain; Cardiology Service, University Hospital La Paz, Madrid, Spain; Nephrology Service, Hospital Universitario Central de Asturias, Oviedo, Spain; Primary Care Center Cartagena Casco, Cartagena, Murcia, Spain; Biomedical Research Institute of Murcia (IMIB), Murcia, Spain; Nephrology Service, University Hospital Infanta Leonor, Madrid, Spain

To the Editor,

Chronic kidney disease (CKD) is frequently identifiable from routine laboratory data, yet under-recording in electronic health records (EHRs) may delay implementation of guideline-based kidney-protective care [1,2]. We analysed 5-year trends in CKD burden and diagnosis recording in Spain using BIG-PAC real-world data from two cross-sectional cohorts of adults with at least one estimated glomerular filtration rate (eGFR) and one urine albumin–creatinine ratio (UACR) measurement near 1 January 2018 and 1 January 2023.

In 2023, 80 919 individuals met inclusion criteria, compared with 70 973 in 2018. CKD, defined by diagnostic coding and/or laboratory criteria, was present in 31.5% of the 2023 cohort versus 30.6% in 2018, confirming the substantial burden of CKD previously reported in the same Spanish database [3]. Despite this, diagnosis recording remained poor. In 2018, among 21 715 individuals with CKD, 6999 (32.23%) had a recorded CKD diagnosis and 14 716 (67.77%) were unrecorded. In 2023, among 25 689 individuals with CKD, 9234 (35.95%) had a recorded diagnosis and 16 455 (64.05%) remained unrecorded. Thus, coding improved only modestly over time, while nearly two-thirds of CKD cases remained undocumented in routine clinical records (Table 1).

**Table 1:** Trends in CKD burden, diagnosis recording, and selected kidney–cardiovascular therapies in Spain, 2018 and 2023.

	Cohort 2018	Cohort 2023
Total population, *n*	70973	80919
No CKD, G1-2/A1, *n* (%)	49258 (69.4)	55230 (68.25)
CKD population, *n* (%)	21715 (30.6)	25689 (31.5)
Unrecorded CKD, *n* (% of CKD)	14716 (67.77)	16455 (64.05)
Among unrecorded CKD (%)		
A1	20.77	19.34
A2	71.02	72.57
A3	8.21	8.09
G3	32.60	31.13
G3a	26.65	25.89
G3b	5.95	5.23
G4	1.06	0.99
G5 without dialysis	0.12	0.12
Comorbidities among the overall population, *n* (%)		
CVD	14006 (19.7)	17266 (21.3)
T2D	22245 (31.3)	24745 (30.6)
Hypertension	25698 (37)	32303 (40.4)
Kidney–cardiovascular therapies among the overall population, *n* (%)		
RAASi	41048 (57.8)	47696 (58.9)
SGLT2i (in non-T2D)	130 (0.2)	829 (1)
SGLT2i (in T2D)	1499 (2.1)	12391 (15.3)
Statins	32011 (45.1)	38413 (47.5)
Diuretics	19193 (27)	22275 (27.5)

Percentage of unrecorded diagnosis of CKD by KDIGO category (albuminuria A1, A2, A3, eGFR, G3, G4, G5) and of selected comorbidities and medication use in each cohort (index date 1/01/2018 and index date 1/01/2023).

CVD: cardiovascular disease; eGFR: estimated glomerular filtration rate; KDIGO: Kidney Disease: Improving Global Outcomes; RAASi: renin angiotensin aldosterone system inhibitors; SGLT2i: sodium-glucose cotransporter-2 inhibitors; T2D, type two diabetes.

The pattern of unrecorded CKD was consistent across the two cohorts. Most uncoded cases were concentrated in the A2 albuminuria category, accounting for 71.02% of unrecorded CKD in 2018 and 72.57% in 2023, whereas A1 and A3 represented smaller proportions. By kidney function stage, unrecorded CKD clustered mainly in G3, particularly G3a (26.65% in 2018 and 25.89% in 2023), with much lower representation in G4 and G5 without dialysis. This distribution is clinically relevant because the largest coding gap appears in intermediate-risk categories, where timely recognition may still enable earlier intervention and more effective risk reduction.

These findings should be interpreted alongside other results from the same research programme. Previous analyses in this population showed etiological coding remains very limited, particularly in earlier stages [3]. The broader 2023 analysis by KDIGO categories also showed that, despite the increase in the proportion of patients classified in moderate, high, and very high KDIGO risk categories, and the growing burden of cardiovascular disease and hypertension, there was only discrete improvements in the use of guideline-directed therapies, with continued underuse of kidney-protective treatment across KDIGO categories (Table 1 and Supplementary Material). Taken together, these data suggest that the gap between laboratory evidence of CKD and formal diagnostic recognition remains a major barrier to structured management.

This has important clinical implications. A recorded CKD diagnosis increases disease visibility within the healthcare system and may facilitate risk stratification, follow-up, implementation of nephroprotective therapy, and coordination across specialties [4,6]. Conversely, failure to code CKD may contribute to therapeutic inertia and missed opportunities for cardiovascular and renal risk reduction [4,5]. This is particularly relevant because recent evidence from the same dataset suggests that CKD is also associated with increasing treatment complexity and polypharmacy beyond the effect of chronological age alone.

In summary, this real-world analysis shows that CKD burden increased slightly in Spain between 2018 and 2023, whereas improvement in diagnosis coding was modest. In 2023, nearly two-thirds of individuals meeting CKD criteria still lacked a recorded diagnosis, with uncoded cases clustering mainly in A2 and G3 categories. These findings support more systematic laboratory-based CKD phenotyping, EHR coding prompts, and integrated care pathways to close the gap between CKD detection and clinical action.

## Supplementary Material

sfag213_Supplemental_File

## Data Availability

Data supporting this study is available from the BIG-PAC database upon reasonable requests.
