# Supplementary material for: Trends in the management and coding of chronic kidney disease in Spain: cross-sectional analyses of real-life data with a 5-year interval
Source: Clin Kidney J. 2026 Jun 24;19(7):sfag213. doi: 10.1093/ckj/sfag213 (PMC13370825; doi:10.1093/ckj/sfag213)
Supplement: sfag213_Supplemental_File [file sfag213_supplemental_file.docx]

**Supplementary table 1.** Baseline characteristics of the overall population in cohort 2 (index 1/01/2023).

| **Overall (N=80919; 100%)** | |
| --- | --- |
| **Biodemographic data** | |
| Age (years), mean (SD) | 64.88 (15.54) |
| Gender (female), n (%) | 39723 (49.1) |
| BMI, kg/m2, mean (SD) | 27.68 (6.37) |
| SBP, mmHg, mean (SD) | 128.32 (22.1) |
| DBP, mmHg, mean (SD) | 75.68 (11.05) |
| **Comorbidities, n (%)** | |
| CVD, n (%) | 17266 (21.3) |
| Coronary ischemic disease, n (%) | 5801 (7.2) |
| Heart failure, n (%) | 5370 (6.6) |
| Stroke, n (%) | 2750 (3.4) |
| Peripheral artery disease, n (%) | 4226 (5.2) |
| Diabetes, n (%) | 25302 (31.3) |
| Type 1 diabetes, n (%) | 557 (0.7) |
| Type 2 diabetes, n (%) | 24745 (30.6) |
| Hypertension, n(%) | 32303 (40.4) |
| No CVD nor Diabetes, n (%) | 46822 (57.9) |
| **Kidney-Cardiovascular therapies, n (%)** | |
| RAASi, n (%) | 47696 (58.9) |
| ACEi, n (%) | 25446 (31.4) |
| At maximal doses, n (%) | 13762 (54.1) |
| ARBs, n (%) | 23631 (29.2) |
| At maximal doses, n (%) | 12807 (54.2) |
| MRAs, n (%) | 1543 (1.9) |
| ARNI, n (%) | 363 (0.4) |
| SGLT2i (in non-T2DM patients), n (%) | 829 (1.0) |
| SGLT2i (in T2DM patients), n (%) | 12391 (15.3) |
| Beta blockers, n (%) | 12927 (16.0) |
| Diuretics, n (%) | 22275 (27.5) |
| Calcium channel blockers, n (%) | 9125 (11.3) |
| Statins, n (%) | 38413 (47.5) |
| **Diabetes medication, n (% within T2DM patients)** | 25189 (99.6) |
| Metformin, n (%) | 17928 (70.9) |
| SU, n (%) | 3586 (14.2) |
| DPP4i, n (%) | 7812 (30.9) |
| Metiglinides, n (%) | 17 (0.1) |
| GLP-1 AR, n (%) | 9170 (36.2) |
| Insulin, n (%) | 8199 (32.4) |
| SGLT2i (in T2DM patients), n (%) | 12391 (49.0) |
| **Biochemical parameters** | |
| Creatinine (mg/dL), mean (SD) | 1.01 (0.6) |
| eGFR (ml/min/1.73m2), mean (SD) | 81.02 (21.14) |
| UACR (mg/g), mean (SD) | 72.47 (211.01) |
| Glucose (mg/dL), mean (SD) | 109.73 (17.21) |
| HbA1c (%), mean (SD) | 6.96 (1.1) |
| Total cholesterol (mg/dL), mean (SD) | 192.31 (25.07) |
| HDL-cholesterol (mg/dL), mean (SD) | 49.65 (8.14) |
| LDL-cholesterol (mg/dL), mean (SD) | 116.26 (21.4) |
| Triglycerides (mg/dL), mean (SD) | 136.9 (59.36) |
| Potassium (mmol/L), mean (DE) | 4.99 (0.69) |
| Hemoglobin (g/L), mean (DE) | 14.29 (1.49) |

ACEi:  angiotensin-converting enzyme inhibitors; ARB: angiotensin II receptor blockers; CVD: cardiovascular disease; DBP: diastolic blood pressure; DPP4i: dipeptidyl peptidase-4 inhibitors; eGFR: estimated glomerular filtration rate; GLP-1 AR: glucagon-Like Peptide-1 Receptor Agonists; KDIGO: Kidney Disease: Improving Global Outcomes; MRA: mineralocorticoid receptor antagonists; RAASi: renin angiotensin aldosterone system inhibitors; SBP: systolic blood pressure; SGLT2 i: sodium-glucose cotransporter-2 inhibitors; SU: sulphonylureas; T2DM: type 2 diabetes mellitus; UACR (urine albumin-creatinine ratio): mg/g.

**Supplementary Table 2.** Baseline characteristics and distribution of the population in cohort 2 (index 1/01/2023) by KDIGO category.

|  | **A1 G1**  **(N=27710; 34.2%)** | **A1 G2**  **(N=27520; 34.0%)** | **A1 G3a**  **(N=4148; 5.1%)** | **A1 G3b**  **(N=1746; 2.2%)** | **A1 G4**  **(N=400; 0.5%)** | **A1 G5 (no dialysis) (N=51; 0.1%)** | **A2 G1**  **(N=4167; 5.1%)** | **A2 G2**  **(N=7415; 9.2%)** | **A2 G3a**  **(N=2180; 2.7%)** | **A2 G3b**  **(N=1563; 1.9%)** | **A2 G4**  **(N=661; 0.8%)** | **A2 G5 (no dialysis) (N=51; 0.1%)** |
| --- | --- | --- | --- | --- | --- | --- | --- | --- | --- | --- | --- | --- |
| **Biodemographic data** | | | | | | | | | | | | |
| Age (years), mean (SD) | 52.12 (12.73) | 69.36 (11.17) | 78.21 (9.4) | 82.02 (8.81) | 83.99 (9.36) | 77.03 (13.63) | 58.57 (11.57) | 74.49 (10.59) | 79.44 (9.57) | 82.91 (8.98) | 84.02 (9.15) | 81.51 (10.63) |
| Gender (female), n (%) | 12940 (46.7) | 13837 (50.3) | 2119 (51.1) | 1030 (59.0) | 265 (66.3) | 29 (56.9) | 1886 (45.3) | 3999 (53.9) | 1049 (48.1) | 844 (54.0) | 398 (60.2) | 24 (47.1) |
| BMI, kg/m2, mean (SD) | 26.5 (13.21) | 27.7 (13.38) | 28.47 (4.9) | 28.55 (4.9) | 28.13 (5.31) | 24.36 (5.35) | 29.97 (5.17) | 29.05 (4.8) | 28.5 (4.8) | 28.49 (4.91) | 28.4 (5.33) | 26.05 (5.07) |
| SBP, mmHg, mean (SD) | 123.14 (48.86) | 129.7 (50.38) | 132.85 (17.27) | 132.74 (17.29) | 130.51 (17.71) | 133.47 (12.44) | 130.01 (17.61) | 133.38 (17.47) | 132.42 (16.97) | 133.52 (17.32) | 131.04 (16.93) | 131.59 (12.86) |
| DBP, mmHg, mean (SD) | 76.47 (29.38) | 75.93 (28.73) | 74.14 (8.05) | 71.32 (7.76) | 70.61 (7.84) | 73.04 (6.7) | 78.95 (8.85) | 75.23 (8.57) | 73.68 (8.17) | 71.51 (7.91) | 70.28 (7.94) | 73.27 (6.24) |
| **Comorbidities** | | | | | | | | | | | | |
| CVD, n (%) | 2147 (7.7) | 5364 (19.5) | 1556 (37.5) | 854 (48.9) | 247 (61.8) | 17 (33.3) | 726 (17.4) | 2468 (33.3) | 1027 (47.1) | 879 (56.2) | 383 (57.9) | 32 (62.7) |
| Coronary ischemic disease, n (%) | 838 (3.0) | 1936 (7.0) | 497 (12.0) | 250 (14.3) | 60 (15.0) | 4 (7.8) | 218 (5.2) | 776 (10.5) | 302 (13.9) | 289 (18.5) | 124 (18.8) | 11 (21.6) |
| Heart failure, n (%) | 310 (1.1) | 1238 (4.5) | 541 (13.0) | 412 (23.6) | 151 (37.8) | 8 (15.7) | 170 (4.1) | 780 (10.5) | 379 (17.4) | 451 (28.9) | 210 (31.8) | 18 (35.3) |
| Stroke, n (%) | 334 (1.2) | 816 (3.0) | 262 (6.3) | 133 (7.6) | 38 (9.5) | 5 (9.8) | 109 (2.6) | 443 (6.0) | 187 (8.6) | 132 (8.4) | 57 (8.6) | 5 (9.8) |
| Peripheral artery disease, n (%) | 637 (2.3) | 1113 (4.0) | 331 (8.0) | 200 (11.5) | 54 (13.5) | 2 (3.9) | 245 (5.9) | 581 (7.8) | 275 (12.6) | 214 (13.7) | 87 (13.2) | 4 (7.8) |
| Diabetes, n (%) | 5479 (19.8) | 8359 (30.4) | 1442 (34.8) | 684 (39.2) | 156 (39.0) | 23 (45.1) | 1781 (42.7) | 3367 (45.4) | 1079 (49.5) | 782 (50.0) | 346 (52.3) | 27 (52.9) |
| Type 1 diabetes, n (%) | 205 (0.7) | 108 (0.4) | 16 (0.4) | 7 (0.4) | 1 (0.3) | 1 (2.0) | 54 (1.3) | 49 (0.7) | 16 (0.7) | 12 (0.8) | 8 (1.2) | 0 (0.0) |
| Type 2 diabetes, n (%) | 5274 19.0) | 8251 (30.0) | 1426 (34.4) | 677 (38.8) | 155 (38.8) | 22 (43.1) | 1727 (41.4) | 3318 (44.7) | 1063 (48.8) | 770 (49.3) | 338 (51.1) | 27 (52.9) |
| Hypertension, n (%) | 6448 (23.3) | 10999 (40) | 2455 (59.2) | 1195 (68.4) | 316 (79) | 41 (80.4) | 1662 (39.9) | 4029 (54.3) | 1561 (71.6) | 1238 (79.2) | 594 (89.9) | 43 (84.3) |
| No CVD nor diabetes, n (%) | 21115 (76.2) | 16449 (59.8) | 1721 (41.5) | 556 (31.8) | 100 (25) | 22 (43.1) | 2031 (48.7) | 2801 (37.8) | 606 (27.8) | 348 (22.3) | 128 (19.4) | 13 (25.5) |
| **Kidney-Cardiovascular therapies** | | | | | | | | | | | | |
| RAASi, n (%) | 11763 (42.5) | 16765 (60.9) | 3292 (79.4) | 1373 (78.6) | 289 (72.3) | 30 (58.8) | 2709 (65.0) | 5541 (74.7) | 1766 (81.0) | 1240 (79.3) | 493 (74.6) | 30 (58.8) |
| ACEi, n (%) | 7033 (25.4) | 8749 (31.8) | 1682 (40.5) | 629 (36.0) | 135 (33.8) | 11 (21.6) | 1636 (39.3) | 2936 (39.6) | 870 (39.9) | 531 (34.0) | 181 (27.4) | 11 (21.6) |
| At maximal doses, n (%) | 3060 (43.5) | 3745 (42.8) | 749 (44.5) | 285 (45.3) | 68 (50.4) | 6 (54.5) | 1341 (82.0) | 2388 (81.3) | 716 (82.3) | 438 (82.5) | 150 (82.9) | 9 (81.8) |
| ARBs, n (%) | 4750 (17.1) | 8045 (29.2) | 1860 (44.8) | 833 (47.7) | 160 (40.0) | 21 (41.2) | 1243 (29.8) | 2998 (40.4) | 1031 (47.3) | 795 (50.9) | 325 (49.2) | 21 (41.2) |
| At maximal doses, n (%) | 1969 (41.5) | 3233 (40.2) | 785 (42.2) | 328 (39.4) | 56 (35.0) | 2 (9.5) | 1021 (82.1) | 2481 (82.8) | 847 (82.2) | 659 (82.9) | 266 (81.8) | 16 (76.2) |
| MRAs, n (%) | 99 (0.4¡ | 301 (1.1) | 167 (4.0) | 104 (6.0) | 24 (6.0) | 2 (3.9) | 99 (2.4) | 253 (3.4) | 123 (5.6) | 124 (7.9) | 45 (6.8) | 1 (2.0) |
| ARNI, n (%) | 4 (0.0) | 11 (0.0) | 44 (1.1) | 26 (1.5) | 3 (0.8) | 1 (2.0) | 29 (0.7) | 86 (1.2) | 42 (1.9) | 49 (3.1) | 10 (1.5) | 0 (0.0) |
| SGLT2i (in non-T2DM patients), n (%) | 21 (0.1) | 56 (0.2) | 119 (2.9) | 42 (2.4) | 16 (4.0) | 3 (5.9) | 80 (1.9) | 205 (2.8) | 76 (3.5) | 62 (4.0) | 23 (3.5) | 2 (3.9) |
| Beta blockers, n (%) | 1904 (6.9) | 4240 (15.4) | 1115 (26.9) | 559 (32.0) | 144 (36.0) | 12 (23.5) | 566 (13.6) | 1758 (23.7) | 691 (31.7) | 591 (37.8) | 253 (38.3) | 15 (29.4) |
| Diuretics, n (%) | 3641 (13.1) | 9228 (33.5) | 1656 (39.9) | 804 (46.0) | 213 (53.3) | 15 (29.4) | 837 (20.1) | 2516 (33.9) | 920 (42.2) | 770 (49.3) | 341 (51.6) | 26 (51.0) |
| Calcium channel blockers, n (%) | 1304 (4.7) | 2741 (10.0) | 649 (15.6) | 387 (22.2) | 88 (22.0) | 8 (15.7) | 531 (12.7) | 1292 (17.4) | 503 (23.1) | 391 (25.0) | 217 (32.8) | 16 (31.4) |
| Statins, n (%) | 9845 (35.5) | 13307 (48.4) | 2565 (61.8) | 1089 (62.4) | 236 (59.0) | 26 (51.0) | 2123 (50.9) | 4468 (60.3) | 1376 (63.1) | 988 (63.2) | 397 (60.1) | 16 (31.4) |
| **Diabetes medications, n (% in diabetic population)** | 5473 (99.9) | 8333 (99.7) | 1427 (99.0) | 676 (98.8) | 156 (100.0) | 23 (100.0) | 1767 (99.2) | 3358 (99.7) | 1065 (98.7) | 771 (98.6) | 346 (100.0) | 27 (100.0) |
| Metformin, n (%) | 4241 (77.4) | 4375 (52.3) | 1256 (87.1) | 508 (74.3) | 36 (23.1) | 2 (8.7) | 1570 (88.2) | 3248 (96.5) | 921 (85.4) | 584 (74.7) | 44 (12.7) | 4 (14.8) |
| SU, n (%) | 576 (10.5) | 1221 (14.6) | 256 (17.8) | 74 (10.8) | 11 (7.1) | 1 (4.3) | 298 (16.7) | 685 (20.3) | 161 (14.9) | 93 (11.9) | 20 (5.8) | 5 (18.5) |
| DPP4i, n (%) | 290 (5.3) | 1250 (15.0) | 781 (54.2) | 506 (74.0) | 139 (89.1) | 15 (65.2) | 733 (41.2) | 1554 (46.2) | 607 (56.3) | 572 (73.1) | 319 (92.2) | 17 (63.0) |
| Metiglinides, n (%) | 0 (0.0) | 0 (0.0) | 4 (0.3) | 1 (0.1) | 0 (0.0) | 0 (0.0) | 4 (0.2) | 1 (0.0) | 1 (0.1) | 1 (0.1) | 1 (0.3) | 0 (0.0) |
| GLP-1 AR, n (%) | 3457 (63.1) | 3438 (41.1) | 399 (27.7) | 137 (20.0) | 26 (16.7) | 1 (4.3) | 446 (25.0) | 777 (23.1) | 171 (15.8) | 79 (10.1) | 25 (7.2) | 1 (3.7) |
| Insulin, n (%) | 1362 (24.9) | 2509 (30.0) | 582 (40.4) | 278 (40.6) | 112 (71.8) | 14 (60.9) | 434 (24.4) | 1043 (31.0) | 412 (38.2) | 296 (37.9) | 187 (54.0) | 18 (66.7) |
| SGLT2i (in T2DM patients), n (%) | 3767 (68.8) | 4580 (54.8) | 778 (54.0) | 294 (43.0) | 27 (17.3) | 1 (4.3) | 567 (31.8) | 1207 (35.8) | 402 (37.3) | 267 (34.1) | 66 (19.1) | 2 (7.4) |
| **Biochemical parameters** | | | | | | | | | | | | |
| Creatinine (mg/dL), mean (SD) | 0.65 (0.27) | 0.98 (0.39) | 1.46 (0.1) | 1.93 (0.14) | 2.89 (0.4) | 6.03 (0.97) | 0.7 (0.1) | 1.03 (0.12) | 1.46 (0.1) | 1.93 (0.14) | 2.9 (0.38) | 5.86 (0.92) |
| eGFR (ml/min/1.73m2), mean (SD) | 99.15 (4.27) | 79.13 (7.92) | 52.28 (4.32) | 37.54 (4.33) | 22.79 (4.38) | 7.84 (4.16) | 102.64 (7.27) | 74.82 (8.68) | 52.41 (4.36) | 37.61 (4.34) | 22.28 (4.26) | 7.87 (4.09) |
| UACR (mg/g), mean (SD) | 9.09 (5.13) | 11.27 (6.47) | 14.39 (8.12) | 17.01 (9.09) | 16.73 (9.8) | 19.94 (11.07) | 130.93 (61.84) | 148 (69.68) | 164.05 (77.48) | 181.98 (81.36) | 191.13 (82.73) | 220.7 (77.5) |
| Glucose (mg/dL), mean (SD) | 102.11 (14.75) | 108.71 (15.36) | 115.19 (15.03) | 117.32 (15.85) | 119.52 (17.53) | 117.14 (23.27) | 122.37 (18.74) | 121.67 (17.28) | 115.86 (15.1) | 117.8 (15.49) | 117.86 (17.46) | 110.45 (25.85) |
| HbA1c (%), mean (SD) | 7.02 (2.84) | 6.99 (3.27) | 6.77 (0.9) | 6.84 (0.87) | 6.89 (1.04) | 6.6 (0.93) | 7 (1.11) | 6.98 (1) | 6.77 (0.88) | 6.82 (0.93) | 7.04 (1.04) | 6.77 (1.01) |
| Total cholesterol (mg/dL), mean (SD) | 197.01 (43.58) | 194.85 (46.66) | 181.95 (20.51) | 179.04 (21.64) | 176.82 (22.7) | 181.7 (21.86) | 193.2 (21.66) | 185.9 (21.19) | 182.41 (20.15) | 180.16 (21.3) | 175.31 (22.63) | 177.77 (20.44) |
| HDL-cholesterol (mg/dL), mean (SD) | 50.61 (8.57) | 50.75 (8.44) | 47.3 (6.42) | 45.82 (6.44) | 44.44 (6.99) | 43.6 (7.45) | 47.78 (6.5) | 48.58 (6.66) | 47.25 (6.42) | 45.72 (6.49) | 44 (6.9) | 45.23 (6.69) |
| LDL-cholesterol (mg/dL), mean (SD) | 121.24 (21.9) | 118.13 (21.84) | 106.83 (17.41) | 104.12 (17.47) | 103.37 (17.81) | 105.77 (18.54) | 116.2 (17.98) | 109.69 (17.45) | 106.79 (16.81) | 104.52 (17.61) | 102.68 (17.48) | 109.55 (20.85) |
| Triglycerides (mg/dL), mean (SD) | 129.14 (66.21) | 132.25 (53.81) | 146.79 (44.72) | 152.67 (52.74) | 159.06 (51.67) | 166.74 (53.5) | 157.84 (71.99) | 146.12 (51.61) | 147.17 (44.46) | 155.74 (54.62) | 158.45 (49.35) | 152.06 (46.97) |
| Potassium (mmol/L), mean (DE) | 4.93 (0.74) | 4.98 (0.69) | 5.14 (0.78) | 5.16 (0.59) | 5.28 (0.61) | 5.15 (0.58) | 4.93 (0.54) | 5 (0.56) | 5.12 (0.78) | 5.16 (0.59) | 5.34 (0.59) | 5.21 (0.55) |
| Hemoglobin (g/L), mean (DE) | 14.62 (1.41) | 14.45 (1.42) | 13.71 (1.39) | 13.08 (1.46) | 11.71 (1.17) | 11.75 (1.03) | 14.55 (1.18) | 14.09 (1.3) | 13.71 (1.4) | 13.14 (1.44) | 11.79 (1.16) | 11.91 (1.15) |

|  | **A3 G1**  **(N=401; 0.5%)** | **A3 G2**  **(N=813; 1.0%)** | **A3 G3a**  **(N=417; 0.5%)** | **A3 G3b**  **(N=390; 0.5%)** | **A3 G4**  **(N=261; 0.3%)** | **A3 G5 (no dialysis)**  **(N=69; 0.1%)** | **G5D (All dialysis patients)**  **(N=430; 0.5%)** | **Kidney transplant (N=526; 0.7%)** |
| --- | --- | --- | --- | --- | --- | --- | --- | --- |
| **Biodemographic data** | | | | | | | | |
| Age (years), mean (SD) | 58.79 (10.86) | 72.43 (11.24) | 74.65 (11.98) | 79.44 (10.51) | 79.97 (11.86) | 76.69 (12.24) | 69.3 (14.66) | 61.68 (13.11) |
| Gender (female), n (%) | 161 (40.1) | 327 (40.2) | 152 (36.5) | 159 (40.8) | 117 (44.8) | 25 (36.2) | 170 (39.5) | 192 (36.5) |
| BMI, kg/m2, mean (SD) | 29.99 (5.14) | 29.18 (4.74) | 28.96 (4.94) | 28.92 (5) | 28.06 (5.51) | 26.43 (5.77) | 25.2 (6.05) | 23.69 (5.47) |
| SBP, mmHg, mean (SD) | 129.88 (16.89) | 132.83 (17.82) | 133.03 (16.85) | 132.92 (17.79) | 133.5 (16.46) | 130.97 (12.73) | 126.6 (22.48) | 123.36 (21.24) |
| DBP, mmHg, mean (SD) | 79.36 (8.95) | 75.63 (8.38) | 73.99 (7.9) | 71.3 (8) | 70.45 (7.68) | 73.05 (6.61) | 70.38 (10.64) | 73.17 (10.33) |
| **Comorbidities** | | | | | | | | |
| CVD, n (%) | 90 (22.4) | 357 (43.9) | 218 (52.3) | 235 (60.3) | 172 (65.9) | 41 (59.4) | 249 (57.9) | 204 (38.8) |
| Coronary ischemic disease, n (%) | 27 (6.7) | 111 (13.7) | 65 (15.6) | 73 (18.7) | 48 (18.4) | 11 (15.9) | 97 (22.6) | 64 (12.2) |
| Heart failure, n (%) | 28 (7.0) | 145 (17.8) | 92 (22.1) | 102 (26.2) | 112 (42.9) | 23 (33.3) | 115 (26.7) | 85 (16.2) |
| Stroke, n (%) | 15 (3.7) | 47 (5.8) | 40 (9.6) | 41 (10.5) | 22 (8.4) | 8 (11.6) | 33 (7.7) | 23 (4.4) |
| Peripheral artery disease, n (%) | 35 (8.7) | 96 (11.8) | 80 (19.2) | 68 (17.4) | 44 (16.9) | 13 (18.8) | 85 (19.8) | 62 (11.8) |
| Diabetes, n (%) | 220 (54.9) | 483 (59.4) | 256 (61.4) | 254 (65.1) | 144 (55.2) | 46 (66.7) | 181 (42.1) | 193 (36.7) |
| Type 1 diabetes, n (%) | 10 (2.5) | 17 (2.1) | 7 (1.7) | 14 (3.6) | 1 (0.4) | 0 (0.0) | 11 (2.6) | 20 (3.8) |
| Type 2 diabetes, n (%) | 210 (52.4) | 466 (57.3) | 249 (59.7) | 240 (61.5) | 143 (54.8) | 46 (66.7) | 170 (39.5) | 173 (32.9) |
| Hypertension, n (%) | 219 (54.6) | 514 (63.2) | 336 (80.6) | 343 (87.9) | 246 (94.3) | 64 (92.8) |  |  |
| No CVD nor Diabetes, n (%) | 160 (39.9) | 2112 (26.1) | 86 (20.6) | 62 (15.9) | 50 (19.2) | 12 (17.4) |  |  |
| **Kidney-Cardiovascular therapies** | | | | | | | | |
| RAASi, n (%) | 320 (79.8) | 655 (80.6) | 346 (83.0) | 321 (82.3) | 196 (75.1) | 45 (65.2) | 194 (45.1) | 328 (62.4) |
| ACEi, n (%) | 179 (44.6) | 336 (41.3) | 171 (41.0) | 130 (33.3) | 75 (28.7) | 18 (26.1) | 56 (13.0) | 77 (14.6) |
| At maximal doses, n (%) | 144 (80.4) | 265 (78.9) | 138 (80.7) | 110 (84.6) | 61 (81.3) | 16 (88.9) | 32 (57.1) | 41 (53.2) |
| ARBs, n (%) | 165 (41.1) | 395 (48.6) | 224 (53.7) | 220 (56.4) | 132 (50.6) | 29 (42.0) | 136 (31.6) | 248 (47.1) |
| At maximal doses, n (%) | 131 (79.4) | 323 (81.8) | 183 (81.7) | 177 (80.5) | 109 (82.6) | 26 (89.7) | 70 (51.5) | 125 (50.4) |
| MRAs, n (%) | 14 (3.5) | 44 (5.4) | 22 (5.3) | 32 (8.2) | 27 (10.3) | 3 (4.3) | 19 (4.4) | 40 (7.6) |
| ARNI, n (%) | 2 (0.5) | 10 (1.2) | 12 (2.9) | 11 (2.8) | 8 (3.1) | 1 (1.4) | 10 (2.3) | 4 (0.8) |
| SGLT2i (in non-T2DM patients), n (%) | 16 (4.0) | 33 (4.1) | 14 (3.4) | 16 (4.1) | 14 (5.4) | 2 (2.9) | 5 (1.2) | 24 (4.6) |
| Beta blockers, n (%) | 72 (18.0) | 231 (28.4) | 138 (33.1) | 144 (36.9) | 106 (40.6) | 22 (31.9) | 146 (34.0) | 220 (41.8) |
| Diuretics, n (%) | 82 (20.4) | 312 (38.4) | 193 (46.3) | 188 (48.2) | 154 (59.0) | 37 (53.6) | 141 (32.8) | 201 (38.2) |
| Calcium channel blockers, n (%) | 72 (18.0) | 207 (25.5) | 140 (33.6) | 138 (35.4) | 93 (35.6) | 26 (37.7) | 121 (28.1) | 201 (38.2) |
| Statins, n (%) | 226 (56.4) | 518 (63.7) | 259 (62.1) | 244 (62.6) | 167 (64.0) | 36 (52.2) | 232 (54.0) | 295 (56.1) |
| **Diabetes medications (within T2DM patients), n (%)** | 219 (99.5) | 482 (99.8) | 253 (98.8) | 254 (100.0) | 144 (100.0) | 46 (100.0) | 180 (99.4) | 189 (97.9) |
| Metformin, n (%) | 197 (89.5) | 462 (95.7) | 225 (87.9) | 177 (69.7) | 12 (8.3) | 1 (2.2) | 6 (3.3) | 59 (30.6) |
| SU, n (%) | 21 (9.5) | 92 (19.0) | 37 (14.5) | 22 (8.7) | 10 (6.9) | 2 (4.3) | 1 (0.6) | 0 (0.0) |
| DPP4i, n (%) | 89 (40.5) | 200 (41.4) | 151 (59.0) | 184 (72.4) | 124 (86.1) | 34 (73.9) | 127 (70.2) | 120 (62.2) |
| Metiglinides, n (%) | 1 (0.5) | 0 (0.0) | 0 (0.0) | 0 (0.0) | 1 (0.7) | 0 (0.0) | 2 (1.1) | 0 (0.0) |
| GLP-1 AR, n (%) | 39 (17.7) | 62 (12.8) | 26 (10.2) | 17 (6.7) | 5 (3.5) | 1 (2.2) | 58 (32.0) | 5 (2.6) |
| Insulin, n (%) | 73 (33.2) | 199 (41.2) | 120 (46.9) | 125 (49.2) | 82 (56.9) | 26 (56.5) | 178 (98.3) | 149 (77.2) |
| SGLT2i (in T2DM patients), n (%) | 65 (29.5) | 159 (32.9) | 92 (35.9) | 75 (29.5) | 25 (17.4) | 2 (4.3) | 14 (7.7) | 1 (0.5) |
| **Biochemical parameters** | | | | | | | | |
| Creatinine (mg/dL), mean (SD) | 0.69 (0.1) | 1.02 (0.12) | 1.45 (0.1) | 1.92 (0.13) | 2.87 (0.41) | 6.15 (0.93) | 6.99 (1.13) | 1.97 (0.73) |
| eGFR (ml/min/1.73m2), mean (SD) | 102.95 (7.41) | 74.77 (8.7) | 52.26 (4.4) | 37.89 (4.24) | 22.02 (4.52) | 7.06 (4.26) | 12.7 (6.83) | 34.77 (7.58) |
| UACR (mg/g), mean (SD) | 759.81 (302.45) | 882.36 (364.11) | 1092.27 (470.32) | 1267.24 (488.56) | 1367.64 (457.25) | 1706.44 (286.27) | 961.62 (793.22) | 808.37 (812.22) |
| Glucose (mg/dL), mean (SD) | 123.54 (18.59) | 121.91 (17.09) | 114.88 (14.68) | 118.37 (15.97) | 118.21 (17.83) | 111.61 (24.14) | 125.14 (20.61) | 106.29 (11.92) |
| HbA1c (%), mean (SD) | 7.07 (1.07) | 7.08 (0.98) | 6.83 (0.86) | 6.64 (0.94) | 6.87 (1.05) | 6.66 (1.09) | 7.22 (0.96) | 7.2 (0.89) |
| Total cholesterol (mg/dL), mean (SD) | 194.27 (21.9) | 187.3 (22.04) | 183.74 (20.69) | 179.13 (21.69) | 176.68 (22.46) | 177.06 (21.33) | 184.27 (24.15) | 173.19 (22.04) |
| HDL-cholesterol (mg/dL), mean (SD) | 47.79 (6.41) | 48.28 (6.54) | 46.75 (6.34) | 45.93 (6.42) | 43.82 (6.79) | 40.97 (6.06) | 42.96 (6.74) | 47.39 (8.87) |
| LDL-cholesterol (mg/dL), mean (SD) | 116.49 (18.19) | 109.6 (17.38) | 105.62 (17.6) | 103.21 (17.66) | 102.05 (18.89) | 110.24 (19.94) | 108.51 (22.57) | 97.69 (17.37) |
| Triglycerides (mg/dL), mean (SD) | 159.05 (70.31) | 146.76 (52.61) | 148.52 (43.55) | 152.72 (54.09) | 160.71 (53.85) | 167.04 (50.25) | 167.64 (59.24) | 129.01 (33.07) |
| Potassium (mmol/L), mean (DE) | 4.96 (0.55) | 4.98 (0.56) | 5.17 (0.8) | 5.18 (0.58) | 5.26 (0.61) | 5.19 (0.53) | 5.44 (0.64) | 5.22 (0.58) |
| Hemoglobin (g/L), mean (DE) | 14.54 (1.22) | 14.11 (1.28) | 13.72 (1.42) | 13.02 (1.48) | 11.69 (1.09) | 11.9 (1.22) | 11.46 (1.2) | 12.84 (1.44) |

ACEi:  angiotensin-converting enzyme inhibitors; ARB: angiotensin II receptor blockers; CVD: cardiovascular disease; DBP: diastolic blood pressure; DPP4i: d[ipeptidyl peptidase-4 inhibitors;](https://en.wikipedia.org/wiki/Dipeptidyl_peptidase-4_inhibitor) eGFR: estimated glomerular filtration rate; GLP-1 AR: glucagon-Like Peptide-1 Receptor Agonists; KDIGO: Kidney Disease: Improving Global Outcomes; MRA: mineralocorticoid receptor antagonists; RAASi: renin angiotensin aldosterone system inhibitors; SBP: systolic blood pressure; SGLT2 i: sodium-glucose cotransporter-2 inhibitors; SU: sulphonylureas; T2DM: type 2 diabetes mellitus; UACR (urine albumin-creatinine ratio)

**Supplementary table 3.** Distribution of the populations by KDIGO categories for each of the cohorts analyzed in the study. Distribution of the percentages are shown for (A) the overall population, (B) the CKD population and (C) the unrecorded CKD population.

| **2018** | | | | **2023** | | | |
| --- | --- | --- | --- | --- | --- | --- | --- |
| **A.**  Overall study population | | | | | | | |
| **N= 70973** | **A1** | **A2** | **A3** | **N= 80919** | **A1** | **A2** | **A3** |
| **G1 (%)** | 35.13 | 5.1 | 0.5 | **G1 (%)** | 34.2 | 5.1 | 0.5 |
| **G2 (%)** | 34.86 | 9.13 | 1.05 | **G2 (%)** | 34 | 9.2 | 1 |
| **G3a (%)** | 4.96 | 2.67 | 0.51 | **G3a (%)** | 5.1 | 2.7 | 0.5 |
| **G3b (%)** | 2.01 | 1.85 | 0.49 | **G3b (%)** | 2.2 | 1.9 | 0.5 |
| **G4 (%)** | 0.54 | 0.71 | 0.35 | **G4 (%)** | 0.5 | 0.8 | 0.3 |
| **G5 no dialysis (%)** | 0.05 | 0.05 | 0.06 | **G5 no dialysis (%)** | 0.1 | 0.1 | 0.1 |
| **2018** | | | | **2023** | | | |
| **B.** CKD population | | | | | | | |
| **N= 21715 (30.6%)** | **A1** | **A2** | **A3** | **N= 25689 (31.75%)** | **A1** | **A2** | **A3** |
| **G1 (%)** |  | 5.1 | 0.5 | **G1 (%)** |  | 5.1 | 0.5 |
| **G2 (%)** |  | 9.13 | 1.05 | **G2 (%)** |  | 9.2 | 1 |
| **G3a (%)** | 4.96 | 2.67 | 0.51 | **G3a (%)** | 5.1 | 2.7 | 0.5 |
| **G3b (%)** | 2.01 | 1.85 | 0.49 | **G3b (%)** | 2.2 | 1.9 | 0.5 |
| **G4 (%)** | 0.54 | 0.71 | 0.35 | **G4 (%)** | 0.5 | 0.8 | 0.3 |
| **G5 no dialysis (%)** | 0.05 | 0.05 | 0.06 | **G5 no dialysis (%)** | 0.1 | 0.1 | 0.1 |
| **2018** | | | | **2023** | | | |
| **C.** Unrecorded CKD population | | | | | | | |
| **N= 14716 (67.8%)** | **A1** | **A2** | **A3** | **N= 16455 (64.05%)** | **A1** | **A2** | **A3** |
| **G1 (%)** |  | 23.44 | 2.26 | **G1 (%)** |  | 24.16 | 2.31 |
| **G2 (%)** |  | 36.48 | 4.03 | **G2 (%)** |  | 37.34 | 3.96 |
| **G3a (%)** | 16.72 | 8.4 | 1.54 | **G3a (%)** | 16.18 | 8.21 | 1.5 |
| **G3b (%)** | 3.32 | 2.27 | 0.36 | **G3b (%)** | 2.58 | 2.35 | 0.3 |
| **G4 (%)** | 0.67 | 0.39 | 0 | **G4 (%)** | 0.5 | 0.49 | 0.01 |
| **G5 no dialysis (%)** | 0.05 | 0.05 | 0.02 | **G5 no dialysis (%)** | 0.07 | 0.02 | 0.02 |

CKD: chronic kidney disease; KDIGO: Kidney Disease: Improving Global Outcomes.

**Supplementary table 4.** CKD etiologies according to KDIGO categories in the cohort 2.

| **CKD ETIOLOGIES** | **A1 G1**  **(N=27710; 34.2%)** | **A1 G2**  **(N=27520; 34.0%)** | **A1 G3a**  **(N=4148; 5.1%)** | **A1 G3b**  **(N=1746; 2.2%)** | **A1 G4**  **(N=400; 0.5%)** | **A1 G5 (no dialysis) (N=51; 0.1%)** | **A2 G1**  **(N=4167; 5.1%)** | **A2 G2**  **(N=7415; 9.2%)** | **A2 G3a**  **(N=2180; 2.7%)** | **A2 G3b**  **(N=1563; 1.9%)** | **A2 G4**  **(N=661; 0.8%)** | **A2 G5 (no dialysis) (N=51; 0.1%)** | **A3 G1**  **(N=401; 0.5%)** | **A3 G2**  **(N=813; 1.0%)** | **A3 G3a**  **(N=417; 0.5%)** | **A3 G3b**  **(N=390; 0.5%)** | **A3 G4**  **(N=261; 0.3%)** | **A3 G5 (no dialysis)**  **(N=69; 0.1%)** |
| --- | --- | --- | --- | --- | --- | --- | --- | --- | --- | --- | --- | --- | --- | --- | --- | --- | --- | --- |
| None of the following, n (%) | 27447  (99.1) | 27275  (99.1) | 3023  (72.9) | 1079  (61.8) | 125  (31.3) | 10  (19.6) | 3727  (89.4) | 6517  (87.9) | 1591  (73.0) | 934  (59.8) | 164  (24.8) | 8  (15.7) | 355  (88.5) | 707  (87.0) | 320  (76.7) | 258  (66.2) | 64  (24.5) | 10  (14.5) |
| Diabetic, n (%) | 50  (0.2) | 59  (0.2) | 245  (5.9) | 151  (8.6) | 56  (14.0) | 9  (17.6) | 10  (0.2) | 38  (0.5) | 120  (5.5) | 150  (9.6) | 94  (14.2) | 13  (25.5) | 2  (0.5) | 4  (0.5) | 27  (6.5) | 29  (7.4) | 50  (19.2) | 9  (13.0) |
| Hypertensive, n (%) | 54  (0.2) | 30  (0.1) | 165  (4.0) | 106  (6.1) | 51  (12.8) | 3  (5.9) | 22  (0.5) | 69  (0.9) | 96  (4.4) | 116  (7.4) | 86  (13.0) | 7  (13.7) | 1  (0.2) | 8  (1.0) | 20  (4.8) | 23  (5.9) | 31  (11.9) | 4  (5.8) |
| Glomerular diseases, n (%) | 74  (0.3) | 77  (0.3) | 269  (6.5) | 133  (7.6) | 66  (16.5) | 10  (19.6) | 63  (1.5) | 271  (3.7) | 141  (6.5) | 126  (8.1) | 133  (20.1) | 7  (13.7) | 4  (1.0) | 29  (3.6) | 23  (5.5) | 23  (5.9) | 41  (15.7) | 24  (34.8) |
| Hereditary, n (%) | 23  (0.1) | 7  (0.0) | 80  (1.9) | 47  (2.7) | 32  (8.0) | 3  (5.9) | 50  (1.2) | 122  (1.6) | 43  (2.0) | 45  (2.9) | 51  (7.7) | 4  (7.8) | 5  (1.2) | 18  (2.2) | 7  (1.7) | 10  (2.6) | 19  (7.3) | 7  (10.1) |
| Renal tubulo-interstitial diseases, n (%) | 41  (0.1) | 47  (0.2) | 104  (2.5) | 76  (4.4) | 37  (9.3) | 10  (19.6) | 50  (1.2) | 49  (0.7) | 48  (2.2) | 69  (4.4) | 64  (9.7) | 5  (9.8) | 3  (0.7) | 7  (0.9) | 9  (2.2) | 12  (3.1) | 29  (11.1) | 8  (11.6) |
| Miscelaneous, n (%) | 13  (0.0) | 9  (0.0) | 124  (3.0) | 74  (4.2) | 12  (3.0) | 2  (3.9) | 71  (1.7) | 112  (1.5) | 58  (2.7) | 61  (3.9) | 41  (6.2) | 4  (7.8) | 11  (2.7) | 15  (1.8) | 8  (1.9) | 14  (3.6) | 16  (6.1) | 3  (4.3) |
| Other, n (%) | 8  (0.0) | 16  (0.1) | 138  (3.3) | 80  (4.6) | 21  (5.3) | 4  (7.8) | 174  (4.2) | 237  (3.2) | 83  (3.8) | 62  (4.0) | 28  (4.2) | 3  (5.9) | 20  (5.0) | 25  (3.1) | 3  (0.7) | 21  (5.4) | 11  (4.2) | 4  (5.8) |

KDIGO: Kidney Disease: Improving Global Outcomes

**Supplementary Figure 1. Schematic of the study design.**

The overall study population consisted of adult patients with at least one measurement of both eGFR and UACR in local laboratory tests closest to 1^st^ January 2018 (up to 6 months) for cohort 1 and closest to 1^st^ January 2023 (up to 6 months) for cohort 2. The study population was staged according to KDIGO definitions based on eGFR and UACR values at their respective index dates. Baseline characteristics of the study population were collected within one year look-back from their respective index dates. eGFR: estimated glomerular filtration rate; KDIGO: Kidney Disease: Improving Global Outcomes; UACR: urine albumin-creatinine ratio. CKD management consensus by 10 Spanish Scientific societies: García-Maset R, Bover J, Segura de la Morena J, Goicoechea Diezhandino M, Cebollada Del Hoyo J, et al. Information and consensus document for the detection and management of chronic kidney disease. Nefrologia (Engl Ed). 2022;42(3):233-264. doi: 10.1016/j.nefroe.2022.07.003.

**Supplementary Figure 2.** Trends in the proportion of CKD risk categories with time. Distribution of the four CKD risk categories (no risk, moderately increased, high risk and very high) at index 1/01/2023 and the change in the proportions with time are depicted in A. Distribution by KDIGO categories of the percent change in CKD risk categories with time is shown in B.

**Supplementary Figure 3.** Trends in the prevalence of the most frequent comorbidities (A) and related medication use (B) at baseline by KDIGO categories. Connecting segment depicts change between 2018 and 2023 >5 percentage points.

**Supplementary Figure 4.** Trends in the prevalence of cardiovascular diseases, diabetes type 1 and type 2 comorbidities and of the subgroup-no CVD nor diabetes at baseline by KDIGO categories. Connecting segment depicts change between 2018 and 2023 >5 percentage points

**Supplementary Figure 5.** Trends in the prevalence of selected medication use at baseline by KDIGO categories. Connecting segment depicts change between 2018 and 2023 >5 percentage points. ACEi:  angiotensin-converting enzyme inhibitors; ARB: angiotensin II receptor blockers; DPP4i: d[ipeptidyl peptidase-4 inhibitors;](https://en.wikipedia.org/wiki/Dipeptidyl_peptidase-4_inhibitor)  GLP-1 AR: glucagon-Like Peptide-1 Receptor Agonists; KDIGO: Kidney Disease: Improving Global Outcomes; MRA: mineralocorticoid receptor antagonists; SGLT2 i: sodium-glucose cotransporter-2 inhibitors; SU: sulphonylureas; T2DM: type 2 diabetes mellitus.

**Supplementary Figure 5 (continuation).** Trends in the prevalence of selected medication use at baseline by KDIGO categories. Connecting segment depicts change between 2018 and 2023 >5 percentage points

**Supplementary Figure 5 (continuation).** Trends in the prevalence of selected medication use at baseline by KDIGO categories. Connecting segment depicts change between 2018 and 2023 >5 percentage points
